# Position Statement: Minimal Criteria for Reporting Veterinary and Animal Medicine Research for Mesenchymal Stromal/Stem Cells in Orthopedic Applications

**DOI:** 10.3389/fvets.2022.817041

**Published:** 2022-03-07

**Authors:** Debbie J. Guest, Jayesh Dudhia, Roger K. W. Smith, Scott J. Roberts, Michael Conzemius, John F. Innes, Lisa A. Fortier, Richard L. Meeson

**Affiliations:** ^1^Royal Veterinary College, London, United Kingdom; ^2^College of Veterinary Medicine, University of Minnesota, Saint Paul, MN, United States; ^3^CVS Group plc, ChesterGates Veterinary Specialists, Chester, United Kingdom; ^4^Cornell University College of Veterinary Medicine, Ithaca, NY, United States

**Keywords:** mesenchymal, stem, stromal, cell, orthopedic, dog, horse

## Introduction

Mesenchymal stem/stromal cells (MSCs) have become a popular therapeutic strategy after their introduction into equine clinical practice for the treatment of tendon injuries in the early 2000's (1). They have since been used to treat other orthopedic diseases in the horse and, more recently, small companion animals where they have also been investigated for the treatment of neurological and cardiac abnormalities. Domestic mammals provide an ideal “proving ground” for this technology prior to its widespread use in human beings because of the aetiopathological similarities with equivalent human diseases. However, these investigations have involved the use of a wide range of cell products for both research and clinical use which are often poorly characterized and poorly described, with variable outcome descriptors, making objective assessment of safety and efficacy difficult, and comparison between studies impossible. While this was not unexpected when the technology was in early development, much evidence on the nature and function of MSCs has been obtained over the last 20 years. Despite this, there continues to be poorly standardized studies that lack scientific rigor and result in skepticism amongst clinicians regarding MSCs as a clinical therapy that impedes progress in this field. As such, there is an unmet need to standardize key parameters relating to clinical stem cell research in animals to improve the quality of the research reporting and provide greater confidence in the technology.

**Figure 1:**
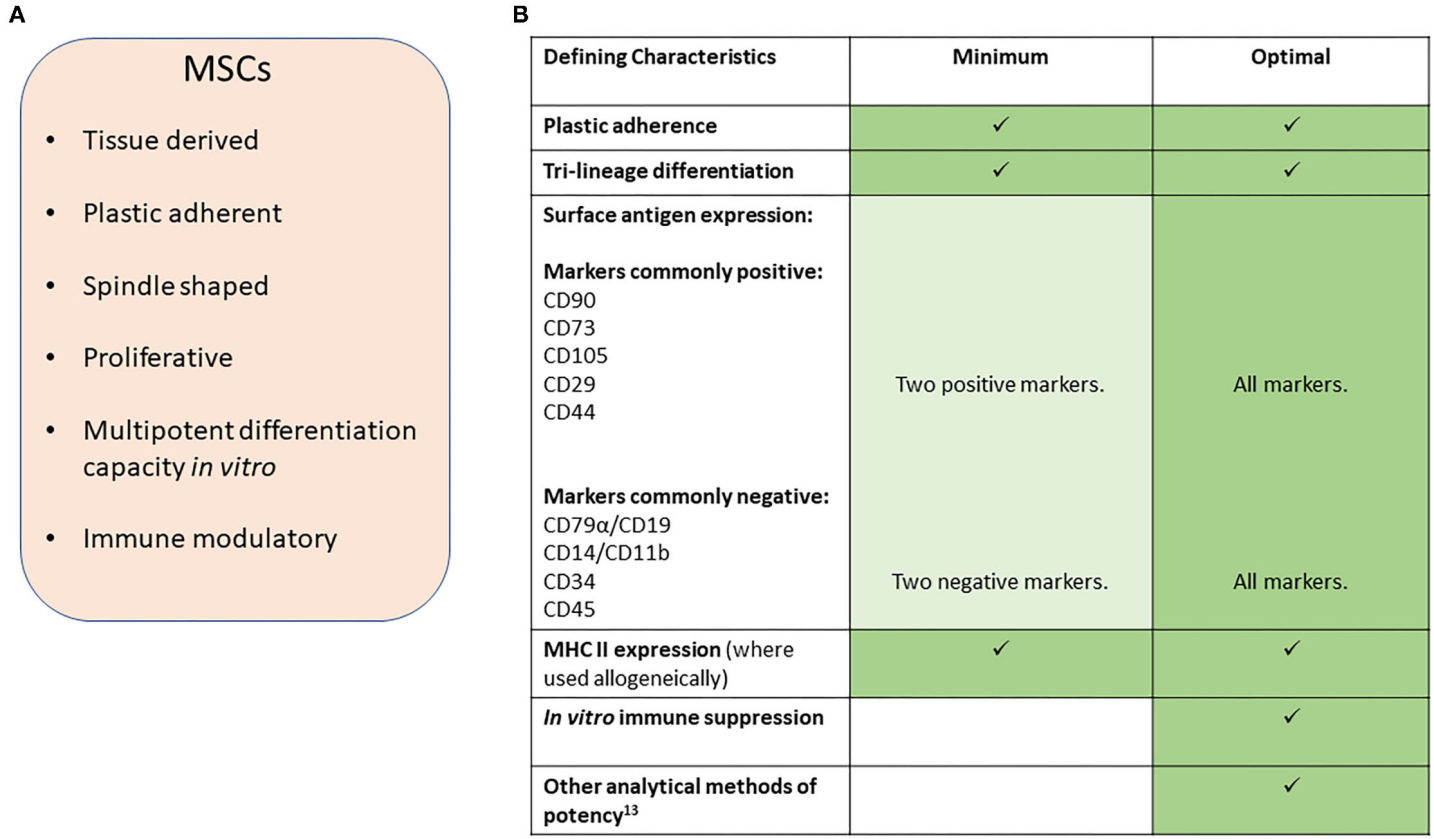
**(A)** Characteristics of MSCs. **(B)** Suggested minimal and optimal definitions of equine and canine MSCs.

In this position statement, we aim to provide guidelines for reporting research involving the use of MSCs in veterinary settings of research and clinical therapy. We have focused on orthopedic conditions in horses and dogs, where the majority of research has been performed, and define MSCs according to the characteristics set out in Figure 1A and similar to the definition used in the human MSC arena (2).

## Defining the Cells

We propose that there should be minimal definition criteria for the identity of the cells based on cell characteristics. However, we recognize that for autologous cells being used in clinical applications it is impractical to fully characterize cells from every case (patient). Even within the research field it is unlikely that laboratories will have the resources to characterize every population of cells which they derive, and there are practical limitations to performing every characterization descriptor while maintaining cells at a low passage number/population doublings for the experiment under investigation. We therefore propose that individual research laboratories or groups follow accepted (published) protocols for the production of MSCs which are described in sufficient detail so that they can be reproduced, or referred to such a description in an appropriate publication. In addition, batch testing of cells being used for research and for any use of allogeneic cells in clinical applications should then be performed to ensure consistency and quality control. As a minimum this should be performed the first time a protocol is applied or whenever a protocol is changed.

Minimal criteria for defining human MSCs, set out by the ISCT (International Society for Cellular Therapy), are in widespread use and include plastic adherence, specific protein surface antigen expression and multipotent differentiation potential (2). However, since these criteria were defined, our understanding of the *in vivo* mechanisms of action of adipose and bone marrow derived-MSCs, as commonly isolated and applied in veterinary protocols, have shifted away from the belief that they will undergo direct differentiation into tissue rebuilding cells, to the demonstration that these cells produce a multitude of factors that have immune suppression and trophic effects and confusion as to the identity of MSCs remains in all species (3–5).

In veterinary medicine, early publications struggled to report on all the criteria set out by the ISCT. This was mainly due to a lack of cross-reacting antibodies to the equivalent cell surface antigen. However, multiple markers for MSCs in dogs and horses have now been reported and species-specific antibodies are now more widely available commercially, although there are still conflicting reports on the exact expression profiles for many of these cell markers. We therefore propose that, a minimum number of species-specific positive and negative markers are used (Figure 1B) with the percentage of positive cells reported. However, we point out that the expression of these markers may vary between species. For example, CD73 is expressed by human (2) and canine MSCs (6, 7) but not equine MSCs (8–10). Furthermore, the culture method used (11) and origin of the cells (5, 12) may affect expression and so must be reported along with the heterogeneity of marker expression within the cell population and between donor animals. This will help to better understand the expression profiles in the horse and dog to build toward a stronger consensus on the most appropriate combination of surface markers to examine than is currently available. In all reports evidence of specific cross-reactivity of the antibodies to the species being investigated must be provided or referenced.

To date, no *in vitro* potency assay to predict MSC efficacy *in vivo* has been defined (13). Furthermore, different assays may be required to predict efficacy for different clinical contexts. No specific marker has been identified which accurately reflects the immunomodulatory abilities of MSCs and *in vitro* read outs commonly involve peripheral blood mononuclear cell proliferation suppression assays. We therefore suggest that, at the present time, an optimal characterization of MSCs would involve the use of *in vitro* immune suppression assays in addition to a tri-lineage differentiation assay [to bone, cartilage and fat, assessed using staining of the cultured cells (2) with quantification performed through dye leaching]. Even though the mechanism of action of MSCs is no longer believed to be through direct differentiation of MSC into tissue cells, the tri-lineage differentiation assay provides a marker of stemness of the MSC.

In addition to these characteristics, and similar to guidelines proposed for human cell therapies (14) publications using veterinary MSCs should state:

- The tissue source for the recovery of the MSCs.
- Preparation method (e.g. enriched or minimally manipulated).
- Culture method (including passaging method, complete media formulation and type/source of serum).
- Passage number and cell seeding densities.
- Method for banking of research and clinically applied cells (15) (total cell number and concentration, passage, cryopreservation media, freezing conditions).

Furthermore, papers using MSCs for *in vivo* applications should state:

- Whether autologous or allogenic cells are used.
- ANTIGENICITY where allogenic cells are used (e.g., MHC expression).
- Cell dose.
- Dosing schedule.
- The vehicle used to suspend and deliver the cells into the animal.
- The method of delivery.
- Other medications delivered with the cells.

The vehicle itself may have independent or co-dependent effects on the impact of the MSCs. Given this, experimental studies should have a control group treated with the vehicle alone and studies based on clinical cases should have a robust comparison control group if possible. Furthermore, where biological products are used to deliver the cells the preparation methods must also be described in enough detail that the methodology can be accurately repeated. For example, platelet rich plasma (PRP) is commonly used as a delivery vehicle, but different preparation methods, as is common in the literature, can result in significant heterogeneity of the final product that is injected into the animal (16).

## Reporting Outcomes

We propose key inclusion and exclusion criteria combined with objective outcome measures that relate to specific mechanisms identified for MSCs in clinical orthopedic studies. Outcome measures have been highly variable between different veterinary studies. We propose obligatory usage of the now well established inclusion criteria and outcome definitions as proposed by Cook et al. (17) shown below. Although some aspects of definitions used here may be debated, the critical issue is that universal application of their definitions allows for an improved comparison between different studies.

### Inclusion Criteria

Clinical research inherently has a variety of clinical variables, and highly specified and narrow inclusion criteria may prevent completion of a veterinary clinical study. It is however important that those variables should be presented in the paper to allow further interrogation of results, particularly for “outliers” as necessary. Minimum recording should include the patient signalment and disease characterization as follows:

- Details of treatment focus: which joint, tendon or ligament.
- Pre-treatment disease state (severity and duration prior to enrollment) and methods that were used to make the assessment.
- Medications/concurrent therapy.
- We recommend inclusion of a clinical summary table, documenting any change in medication/other unexpected events, against summary outcome result for each enrolled animal. This would allow readers to interrogate individual animal results or outliers.

### Disease and Outcome Assessments Measures

Disease status assessment needs to be matched and aligned to treatment outcome assessments. We do not arbitrarily recommend specific assessment criteria as they need to be appropriately tailored to the disease in question, however, wherever possible an objective measure is preferred. They may include one or several of the following categories and ideally applied in combination, as no single assessment is fully comprehensive when evaluating the complex aspects of structural disease, functional limb usage and assessment of pain.

*Imaging assessments*: should involve the most relevant imaging modality and include lesion measurements, or disease grading such as osteoarthritis scoring systems. Wherever possible a recognized scoring system (18) or method of lesion measurement should be made.

*Functional assessments:* are ideally objective such as kinetic (force plate, or the more clinically accessible pressure mats), and kinematic (activity monitors/accelerometers), which can maximize objectivity and minimize care-giver placebo influence. Historically, assessment methods frequently include subjective clinician assessments of visual lameness scores, which have been proven to be unreliable. Goniometry and measures of muscle volume can also be considered if their methodology of measurement is accurately described and consistent.

*Client reported outcome measure assessments (CROMS):* patient assessments have become an integral part of human clinical outcome assessment and certain ones are recognized by the FDA and EMA and are included in some human phase III clinical trials. Although CROMs are semi-objective and there is wider variance compared to gait analysis, there are several studies in dogs now showing that gait analysis and CROMs show the same outcome in trials. Study design is important and care-giver placebo effect needs to be considered which is not an issue with gait analysis, however they can provide measures of the “disease construct” which is not simply identified by gait/locomotion change. Examples include LOAD (Liverpool Osteoarthritis in Dogs) (18), CBPI (Canine Brief Pain Inventory) (19), COI (Canine Orthopedic index) (20), HCPI (Helsinki Chronic Pain Index) (21).

However, these assessment measures should be integrated to allow a global assessment of outcome as per Cook et al. (17) with modifications:

- Full function—restoration to, or maintenance of, full intended level and duration of activities and performance from preinjury or pre-disease status (without medication). This should equate to low scores on clinical metrology questionnaires, indicating negligible identification of pain or impact on quality of life.
- Acceptable function—restoration to, or maintenance of, intended activities and performance from preinjury. This should equate to intermediate scores on clinical metrology questionnaires, indicating presence of some pain and some impact on quality of life.
- Unacceptable function—all other outcomes (such as persistent lameness, reinjury, retirement, or euthanasia because of the disease), with high scores on clinical metrology questionnaires indicative of significant pain and poor quality of life.

When reporting clinical outcomes it is important to record:

- Whether the assessor was blinded to treatment.
- Who the assessors were (notional acknowledgment of their experience; veterinary graduate, new, experienced, advanced or specialist) and how many assessors took part.
- The pre-treatment state using an assessment measure which will also be used to assess response to treatment. i.e., how severely painful or lame, or size of tendon defect prior to treatment.

### Reporting Time Frames

Most studies to-date have had varied follow up periods from 30 days through to two years for both dogs and horses. We endorse the study period terminology established by Cook et al. as follows:

- Perioperative (pre, intra, and postoperative)−0–3 months:
- Short term—>3–6 months.
- Mid term—>6–12 months.
- Long term—>12 months.

Currently many studies fall within the perioperative definition and we encourage more studies to extend into the short to mid-term, but acknowledge the difficulty and loss to follow-up seen in clinical studies as durations increase.

If studies have been carried out in accordance with the criteria we propose then the study outcomes will help to fill our knowledge gap irrespective of the duration of study. Nevertheless, longer periods of follow up clearly offer better scientific evidence of outcomes, particularly if the follow up is carried out periodically over time, as studies measuring the long term efficacy of the cells in improving clinical outcome are rare.

## Concluding Remarks

We propose that the aforementioned framework be applied as our understanding and application of other technologies to this field develops. The markers commonly used to define MSCs do not readily differentiate them from fibroblasts (22), and over time, better combinations or more reliable surface antigens may present themselves. As an example, a Clinical Indications Prediction (CLIP) scale has been developed for human MSCs which uses TWIST1 expression levels to predict the therapeutic efficacy of MSC populations for different disease indications (23). To undertake a similar approach for MSCs in veterinary species would require comparisons of global gene and protein expression data between MSCs and other cell types and there are currently limited datasets available. Over time these approaches will become less cost prohibitive for the veterinary sector and adapting these new approaches does not preclude the continued application of the outlined principles; consistency in reporting, usage of recognized and standardized assessment criteria, and application of universally accepted study definitions.

To conclude, veterinarians and owners still rightly question if and how MSCs will help their pets. We propose that these guidelines should be utilized in publications and presentations to drive higher scientific standards and relevant regulation, and enable better comparison between studies to give greater confidence to the stakeholders of the veterinary field.

## Author Contributions

RM conceived the paper. DG and RM wrote the first draft of the manuscript. All authors contributed to manuscript revision and read and approved the submitted version.

## Conflict of Interest

JI is employed by CVS Group plc. With the University of Liverpool, JI is the joint license holder for the LOAD client-reported outcomes measure. The remaining authors declare that the research was conducted in the absence of any commercial or financial relationships that could be construed as a potential conflict of interest.

## Publisher's Note

All claims expressed in this article are solely those of the authors and do not necessarily represent those of their affiliated organizations, or those of the publisher, the editors and the reviewers. Any product that may be evaluated in this article, or claim that may be made by its manufacturer, is not guaranteed or endorsed by the publisher.
